# Vulnerability identified in clinical practice: a qualitative analysis

**DOI:** 10.1186/s12910-019-0416-4

**Published:** 2019-11-27

**Authors:** Laura Sossauer, Mélinée Schindler, Samia Hurst

**Affiliations:** Department of Internal Medicine, Rehabilitation and Geriatrics, Service of General Internal Medicine, Geneva University Hospitals, Rue Gabrielle Perret-Gentil 4, 1205 Genève, Switzerland

**Keywords:** Qualitative research, Healthcare disparities, Healthcare rationing, Physician’s role, Vulnerable populations

## Abstract

**Background:**

Although it is the moral duty of physicians to protect vulnerable patients, there are no data on how vulnerability is perceived in clinical practice. This study explores how physicians classify someone as “vulnerable”.

**Method:**

Thirty-three physicians were initially questioned about resource allocation problems in their work. The results of these interviews were examined with qualitative study software to identify characteristics associated with vulnerability in patients. Data were conceptualized, classified and cross-linked to highlight the major determinants of vulnerability.

The findings revealed the principal factors that make patients vulnerable in clinical practice, according to our definition of vulnerability: *the likelihood of having one’s interests unjustly considered.*

**Results:**

Vulnerability can arise as a result of a mismatch between the characteristics of patients and physicians, the healthcare system, the treatment, or the communication between physicians and patients. Vulnerability appears as a gap between a patient’s needs and the means intended to meet them. Vulnerability can further be the result of doing too little or too much for patients. This result suggests that structures provided by healthcare systems are not as differentiated as they should be to cover all situations. Our initial definition of vulnerability was illustrated and supported by our results, showing that it encompasses all factors involved, not solely personal characteristics, indicating the need for a more pragmatic approach for use in clinical practice.

**Conclusion:**

Vulnerability is not due to a single factor but appears under certain circumstances when there is a discrepancy between a patient’s interests and the care provided, despite existing compensation systems.

## Background

In clinical ethics of healthcare, it is commonly assumed that vulnerable persons or groups deserve special attention, care or protection. The physicians, as stated in many professional codes, have a moral and professional duty to treat patients equitably and protect those who are vulnerable, especially in research [1]. Moreover, there is a debate in medical ethics about the definition of vulnerability [2–4]. Is it a fundamental part of the human condition [5–7] or an individual characteristic that should be afforded special protection [8, 9]? For this research, we used the definition of vulnerability developed by our team [9, 10].

*In healthcare, particularly vulnerable individuals are those who are more likely to have their interests unjustly considered* [10].

This definition recognizes that previous views of vulnerability refer to the complementary components of the same concept with different likelihoods of occurrence; the notion of just a few patients being vulnerable and therefore requiring protection needs to be reconsidered with the view that vulnerability encompasses everyone. Vulnerability is based on claims that have to be satisfied.

In reality, physicians in clinical settings are faced with ethical difficulties and competing values and goals that they are not always able to successfully resolve [11]. They also face resource constraints [12]. Vulnerability is viewed as an increased risk of having one’s interests unjustly considered, and it is particularly likely to appear in situations where resources are limited; competition for resources results in ongoing difficulties and discussions within health systems because of efforts to limit rising costs and allocate resources [13–17]. Situations with scarce resources are interesting contexts in which to explore clinical vulnerability.

As part of a study designed to explore equity in medical practice, we interviewed family doctors (see ‘Recruitment’) who were asked about situations where they were faced with scarce resources. These results were presented in an earlier study [18]. In this secondary analysis, we wanted to explore *which patients are described as vulnerable by clinicians* to assess whether our definition reflects medical reality or whether it should be modified accordingly.

## Methods

### Recruitment

Physicians working in Geneva with previous training as family doctors (internal medicine, paediatrics, geriatrics) were eligible for recruitment if they were clinically active for at least 20% of their time and for at least 1 year. Participants were recruited by a selective sample and snowball technique. Prior to this study, a research assistant independent from the hospital hierarchy informed participants by telephone about the purpose and process of the study. The contacted physicians worked at the Geneva University Hospitals with in- and outpatients and/or in outpatient medical practices. Of the 52 doctors contacted, 38 agreed to participate (response rate of 73%), and 33 interviews were completed, encompassing a large range of years in practice (1.5–35). This study was approved by the Ethics Review Committee of the Geneva University Hospitals. This secondary analysis was within the scope of the initial research question and participant consent. All identifiers were stripped from the data after the primary analysis.

### Data collection

The aim of the initial study was to learn more about the process of resource allocation in order to improve it. A semi-structured interview using open-ended questions was designed by a team of ethicists, sociologists and physicians to explore practical situations where physicians were confronted with equity problems. The interview protocol was tested during several initial meetings and via 3 pilot interviews. Participants were questioned about their experiences with resource allocation and rationing and their strategies in the face of these issues, and they were questioned with practical examples of situations considered equitable or inequitable (see Table 1). Thirty-minute to one-hour interviews were carried out from January to June 2006. Regular debriefing sessions with the research team were held during the data collection process. No repeat interviews were carried out in the initial study. For data analysis, verbatim transcripts of interviews were imported into QSR NUD*IST, version N6 (QSR International, Victoria, Australia) qualitative research software. No field notes were taken during the initial study. The data presented here are identified by the corresponding interview number [Ix].

**Table 1:** Clinical equity semi-structured interview guide

Domains Initial questions Follow-up questions		
Experience in situation of resource allocation	*In certain situations, doctors can be faced with the question of whether or not to use an intervention because resources are limited.* Do you feel that this has happened to you? Do you have an example of a case where pressure on resources has faced you with a difficult choice in clinical practice? Which decisions were made in this situation? What was your role in this decision? What values seemed important to you when this decision was made? To what extent were you satisfied with the decision that was made? Why?	On the basis of which patient characteristics would you say that it is reasonable/unreasonable to continue an expensive treatment? Why? On the basis of which treatment characteristics would you say that it is reasonable/unreasonable to continue an expensive treatment? Why? Should other aspects have been considered? Which ones?
Nature of concern for equity in clinical decisions	*When resources are limited, doctors are forced to think in terms of fairness.* What does ‘to treat a patient fairly/equitably’ mean to you? What about ‘unfairly/inequitably’? Can you give an example of a situation where it was difficult to treat a patient in your practice fairly? In your first example, did the decision seem fair to you? In what way? Did you think in terms of fairness in this first example?	Can you give an example of unfair treatment? Do you have a specific case experienced?
Training	How did your idea of fairness develop? To what degree do you feel prepared by your training to face questions of fairness?	Which parts of your training seem to you to have been useful in helping you face these cases? In your clinical practice, what leads you to question yourself about fairness? Do you think questions of fairness are more present now than before in your practice? What does this mean for your view? And insurance in all this?

*Italic*: General introduction of the original interview

### Data analysis

We adapted the code and analytical elements of grounded theory [19, 20]. Phrases were selected on the theoretical basis that the definition of vulnerability is *being more likely to have one’s interests unjustly considered* to extract the vulnerability factors that were explicitly mentioned.

Similarities in themes, conceptualizations and comparisons were primarily made with N6. Recurring concepts were labelled with a set of codes that grew over time. In the second step, a tree code was built and hierarchized: the initial concepts were reassembled into groups or categories according to the identified relationships and then broadened to capture their different aspects. An example of a vulnerability factor that was explicitly mentioned is: “There are always patients like that; unreliable, untrue, people by whom we are not motivated and who make us sometimes say to ourselves that we want to punish them” (I.27). This quotation, initially coded under “not compliant”, belongs to the patient category “personality”. This category was broadened to include other aspects such as fear or personal beliefs. Finally, we identified the core category “characteristics of patients” that integrated minor themes such as “personality”, “habits” or “socio-demographics” under the same category because vulnerability depends not only on personal traits but also on the social or cultural context.

We also coded situations in which additional measures were needed, indicating that a risk was present, linked to particular characteristics. An example of an indirectly conveyed risk is: “We have a foreigner or marginalized person and I think we try to follow the rule of giving care to everyone” (I.13). Being a foreigner or marginalized individual is indirectly mentioned here in response to a question about equity; even though there is no wrongdoing, the risk of unfair care remains because the status implies additional effort for the physicians.

Early data were re-examined using the final coding system because the codes emerged slowly through the analysis process and the progressive, larger vision of the corpus. Finally, pertinent codes were added by searching with keywords and synonyms to ensure that all concepts were included in the code. The main categories described here reached data saturation, the point where new data did not contribute to any further development of the code [21].

The tree code is presented in Table 2. When the data were sufficiently stable, a common resolution level was decided upon to provide a view of major themes, and sub-branches were included in the major branches to allow comparisons.

**Table 2:** Vulnerability determinants

(No. of interviews mentioned/total)	Examples of illustrating quotations
1.Health-care system (31/33)	
1.1. Hospital Resources (31)	It happens sometimes that we have a problem with the number of beds and we have to make choices. (E10)
1.2. Intrinsic characteristics (compensation systems) (21)	I work with people who do not have insurance and who are working illegally, everything we do outside the Community Medicine Department needs welfare service so it is true that we think very carefully before asking for an examination, especially if it’s expensive. (E12)
1.3. Medical culture (26)	Here in Community Medicine, medical culture is different than internal medicine, we have less recourse to complementary examinations. (E12)
1.4. Actors (18)	In Oregon, they have a budget and the population decided, voted, discussed on what cares are useful or futile or unnecessary. (E24)
2.Physicians (31/33)	
2.1.Knowledge (22)	It’s really by experience, by exchanges with peers, with colleagues that we acquire this reflection and also by seeing what happens in other countries, that we don’t want to see here, I mean inequality in care access. (E25)
2.3.Liberty (6)	By putting a different weight on certain criteria compared with others, this leaves us a big extent of freedom, allowing us to escape pressure in a certain way. (E3)
2.4.Personality (15)	I never refuse care a for patient who needs it, even if he doesn’t pay the bills and we have arrears.(E25)
2.5.Feelings (11)	It happens to always be the same kind of patients who miss their appointments. These are really not reliable and faithful (...) people for who we are not motivated to care and it can happen that sometimes we say to ourselves that we want to punish them. (E27)
2.6.Influenced (27)	There is a reflection for each act, especially when it’s expensive. (E7)
2.7.Professional situation (25)	For example a CT-Scan that I don’t really believe in, but I say to myself: here we are on uncertain ground, it is not completely wrong to do it so that is what I do but in fact it may be excessive. (E13)
3.Treatment (27/33)	
3.1.Heavy side effects (5)	For example a patient with a stroke and for this one we decide not to do an echo-Doppler examination of the neck because if there is an abnormality, we won’t do a surgical intervention. (E1)
3.2.Not repaid (4)	All these infertility problems are only accessible to people who can pay for treatment and not repaid by Swiss social insurances, it’s also an equity problem. (E18)
3.3.No benefits (14)	The main criteria that make me think it’s reasonable to use expensive means is being entitled to expect benefits. (E11)
3.4.Over-interventionism (26)	In private practice where I worked, I was scandalized by the debauchery of technical means without scientific medical justification, it was particularly terrifying. (E11)
3.5.Expensive (26)	It happened to us not to give the dose because it is about one thousand francs for one milligram and we need ten milligrams to treat. (E10)
3.6.Complex (4)	Every patient is different, we could do the same treatment for some of them but other will need more advanced treatment and if we start with this system [globalized care], as it’s going up in hospitals, we won’t be fair because this patient has a more complex problem and that won’t fit the standardized directives. (E25)
3.7.Poly-medication (2)	Biggest limitation in treatments is often the number of drugs because if they already have ten drugs, then we think a lot before introducing an eleventh. (E1)
4.Communication (30/33)	
4.1.Patients’ level of understanding (7)	This patient doesn’t understand... he doesn’t speak our language and anyway we will never manage to explain to him why this is important to do or not to do, this examination or taking this medication, so we forget about it, (…)(E14)
4.2.Physicians-Patients relationship (15)	We feel like doing something differently for someone which seems to us friendly or not friendly, there are many things very subtle operating but in a more individual level I think... I would say that I’m conscious of that but we try to fight against this. (E12)
4.3.Medical explanations (19)	Sometimes we go too far in treating patients; sometimes we treat patients without them understanding the treatment; sometimes we go rapidly to a therapeutic withdrawal, sometimes too rapidly, it’s difficult to know who is right or wrong. (E9)
4.4.Patients’ refusal (16)	I have more the impression of being inequitable if I am not able to give care for someone who needs it but for whatever reason does not want treatment, for social, psycho, psychopathologic reasons. (E22)
5.Patients Characteristics (31/33)	
5.1.Socioeconomic status (24)	We clearly see a part of the rich population becoming more rich, who won’t have care access problems, and a poor population, becoming more poor and having a lot of difficulties of care access. (E10)
5.2.Family (20)	When we [paediatricians] have non-French speaking parents, we spend less time explaining things than when we have a child who comes in and he is the professor’s son who knows everybody then we have to speak to everyone, explaining to everyone, care is different but in the end treatment is the same but expenditure of energy is bigger, now I have many examples in memory. (E18)
5.3.Social environment (21)	With these people I practice medicine but in a more accelerated manner than I would wish because I had to spend time resolving social problems or... care access. (E30)
5.4.Legal status (16)	There are populations for which, for reasons of elevated insurance premiums, fear of identification or legal problems when they are illegals, care is delayed and I think that under certain conditions that could be dangerous for their health. (E12)
5.5.Demographic (29)	Well, we have the reflex to limit treatment in the elderly. (E11)
5.6.Personality (20)	I think that we quickly tend to take cover by saying: well he doesn’t want it, or, for example, he is aggressive, we often hear that in emergencies: ‘When you become polite again, we will try to help you’, that’s wrong, he is aggressive, unpleasant, he is sick and all is probably linked so it’s necessary to help, to make effort to adapt. (E11)
5.7.Culture (21)	They are not treated the same... or when they don’t speak French people take the liberty to do things that when we hear about them later, it’s shocking the way they are cared for or the way they are treated or the way we speak with them, we have many shocking accounts about what is happening with people not from around here. (E13)
5.8.Insurance (25)	If a patient goes to the operating block, if it’s a private patient, he will be operated by the senior attending surgeon, if he has a common insurance, he will be operated by the resident or the junior attending in formation. (E10)
5.9.Medical Characteristics (29)	We say to ourselves given that bad prognostic … comorbidities … reduced life expectancy. We probably won’t invest theses means if we don’t have many available beds. (E3)
5.10.Habits (18)	There is a moral inequity I think in the way that...with poor patients, marginalized, drug addicted, alcoholics, with psychiatric problems, we will probably be without being totally conscious, they have less performing care because... there is less investment. (E13)
5.11.Autonomy (29)	We can also imagine that in front of a patient’s insistence for a treatment... knowing it won’t be useful on the somatic level, it might be efficient on the psychological level. (E11)
6. Mismatches	
6.1.Patients’ characteristics - health-care system	In Africa, when you have resources, you see the professor in public service that refers you to private clinics when you immediately have all the necessary exams you need but patients who cannot pay, will stay in the public system where he may or may not have access to treatment, or it may be too late. (E15)
6.2.Patients’ characteristics - physician ability to communicate	Patients have the right, even in paediatrics, to tell us when they have had enough, even if we think they are too young; although they are minors, they do not decide but they have a say. (E21)
6.3.Patients’ characteristics - treatment characteristics	For certain patients we go far in extremely expensive, heavy and complicated care and we could ask ourselves if it is justified to extend a life by a few months if we allocate resources more efficiently. (E20)
6.4.Patients’ characteristics - physician professional situation	Senior medics attending them (Patients from Emirates) … feel under obligation to propose examinations in their specialty as these patients often have four or five intermediates, each proposing invasive examinations. (E6)

The main themes were cross-linked in a matrix with N6 properties to determine how many interviewees mentioned two codes together, and the frequency of interactions between categories was assessed. An example of a matrix between patients’ and physicians’ characteristics is given in Table 3; the matrix illustrates that the categories “socio-demographic characteristics” and “influence on physician” were simultaneously coded for the same lines in 17 interviews, meaning that this combination of elements was implicitly or explicitly mentioned by physicians as being a risk factor for patients’ interests being unjustly considered. We decided that intersecting codes that occur in ten or more interviews should be included.

**Table 3:** Example of a matrix

	Physicians’ characteristics					
Patients’ characteristics	Background	Freedom	Personality	Feeling towards patient	Under influence	Professional situation
Socio-demographic	9	1	8	2	17	13
Personal	2	0	0	2	5	6
Cultural	3	0	0	1	5	4
Medical	2	0	5	0	13	5
Insurance	2	0	0	0	7	0
Habits	1	0	2	2	6	4
Autonomy	5	0	2	1	10	6

To ensure clarity and dependability during the coding process, we held regular investigator meetings to discuss the coding and current understanding of emerging themes [22]. Ten percent of the data were double-coded and reviewed throughout the process by three researchers to verify that the concepts were clearly defined and that the codes were stable. For the purposes of publication, quotations were translated from the original language, French. The original quotations are available in web annexes.

## Results

### Study participants

The participants’ characteristics are shown in Table 4.

**Table 4:** Participants’ characteristics

	Doctors (*N* = 33)
Characteristics	
Age	29–62
Years in practice	1.5–35
Male/Female	21/12
Speciality	
General/internal medicine	22
Intensive care	4
Paediatrics	5
Geriatrics	2
Primary practice site	
City outpatient	10
Hospital outpatient	11
Inpatient	22
Role	
Resident	7
Junior attending	18
Senior attending	3
Private practice	9

### Determinants of clinical vulnerability

The data suggest that vulnerability depends on five criteria (listed by order of frequency): the patient’s characteristics, physicians in charge of the patients, healthcare system organization, treatment characteristics and communication between physicians and patients. The preceding criteria are described below in Table 2 with explanations, original quotations and frequencies for the themes identified.

### Patient characteristics

The patient’s characteristics could be intrinsic (gender, age), personal (habits, autonomy), medical (state of health, comorbidities), cultural (language, religion) or social (socio-demographics, legal status, insurance). Intrinsic characteristics can lead to prejudice, particularly amongst elderly patients, as it is reported that, in their case, age is used to decide the limits of care. Medical characteristics are often mentioned as being highly subjective. Physicians reported being surprised by the discrepancy between the medical facts on file and reality: for example, information leading them to imagine a patient in very poor general condition and the reality of the person’s condition. Cultural and social characteristics are problematic in accessing care due to a lack of understanding or for networking reasons and because of the physician’s own limitations in establishing contact for time reasons (e.g., solving social problems instead of treating patients) (Table 2/5.3). Socio-economic and socio-demographic characteristics are mostly linked with insurance coverage and legal problems, despite the existence of compensation systems such as small budgets to cover basic examinations. Physicians reported frustration with not being able to treat people as they should as a result of financial resource constraints.

However, characteristics such as financial resources can also lead to overtreatment by offering too much in terms of the means invested. For example, a very rich patient will privately pay for examinations while other patients will not. Very demanding patients can also become vulnerable by leading the physicians to carry out more examinations than necessary to reassure both protagonists.

### Physicians in charge of patients

Physicians can be influenced by their own characteristics, such as background or feelings towards a patient, contextual factors (professional situation), or costs. Their sensitivity to equity problems depends on their personal history, colleagues’ influence and personal experience. Their propensity for charity is suggested to be linked with their personality. Feelings towards patients—including prejudices—may become problematic in terms of treating people with equity due to unpleasant feelings or inappropriate thoughts. Professional conditions, especially lack of time and feeling overwhelmed, might prevent the identification of the patient’s needs. Another example is an unclear medical situation that might also lead to over-interventionism (Table 2/2.7).

Costs also influence physicians’ decisions if they feel responsible towards a patient or society to control costs. High costs of treatment can induce a deeper reflection, which could take the focus away from the patient’s best interests. In contrast, in private practice, higher costs might lead to over-interventionism due to differences in remuneration.

### Healthcare system organization

This category includes insurance, policies and medical culture in hospitals, depending on the medical department. Available resources was frequently mentioned, as well as compensation systems or the remuneration system for medical acts. Health policies can determine care access. Being uninsured, illegal or needing expensive care makes a patient vulnerable when resource access becomes difficult for legal and financial reasons. The compensation systems for these issues are mentioned as being insufficient, leading to alternative medical decisions that are inconsistent with what the situation requires. An uninsured patient may require a special monetary fund that is limited, leading the physician to consider the situation differently from how he or she would consider the situation of an insured patient (Table 2/1.2). Hospital resources can depend on the availability of a given resource or the type of hospital (public or private), although the two are often linked. For example, private clinics are in a position to offer costly treatments. Medical culture, regulated by the nature of the medical department, can also determine resource allocation as well as the mutual influence of peers and collective experience.

### Treatment characteristics

Treatment characteristics encompass the intrinsic properties of treatment (severe side effects, complexity), areas linked with health system organization (price, repayment) and areas linked with the patient (polymedication, no medical benefits). These characteristics can lead to either under- or overtreatment. Cases of under-treatment are linked with expensive or non-reimbursed treatment in cases of financial or insurance problems. Time and energy expenditures are mentioned as needed to settle these problems with compensation systems (including charity), increasing the risk of patients not being cared for as they should. Expensive treatments may influence physicians if they are under external pressure or if they know the patient’s treatment will not be reimbursed. For example, infertility treatments are not reimbursed and available only for patients who can afford it (Table 2/3.2).

Compliance and understanding are often mentioned as limiting access to treatment if the physician has doubts that the treatment will be followed as directed. A treatment’s benefits are subject to interpretation. For example, for elderly or very ill patients, physicians report being less inclined to see the benefits of a treatment.

### Communication between physicians and patients

This category includes the patient’s level of understanding, refusal to receive care, the capacity of the physicians to inform the patients and the quality of the relationship between patients and physicians. The patient’s level of understanding is mentioned in relation to social background (i.e., country of origin, education level) and medical problems that could interfere with treatment (psychiatric or neurological). Capacity to inform is linked with available time, language and complexity of treatment, and the physician’s personality and background. A patient with a low level of comprehension could, for example, induce frustration in the physician who is unable to advise as he or she should, especially when there is little time for explanations, leading the physician to have negative feelings towards the patient. The risk is that the physician will do less for this patient in terms of the time and energy involved. In one interview, a physician mentioned that a patient refusing to follow a course of treatment or advice induces the same feelings of frustration and inequity as poor communication (Table 2/4.4).

### Vulnerability as a mismatch

Cross-linking the data shows that vulnerability appears in the gap between patient characteristics and the categories previously mentioned. Here, the main mismatches found in the matrix are explained, with verbatim examples given in Table 2.

### Patient characteristics and the healthcare system

This gap is mainly determined by socio-economic characteristics and legal status. Physicians mention that this gap is most frequently linked to financial means, difficulty in accessing care, expensive treatments and hospital resources, despite compensation systems.

Examples given include public and private hospitals in a society that lacks basic insurance and huge social disparities, creating a two-tier healthcare system based on the ability to pay (Table 2/6.1).

### Patient characteristics and the physician’s ability to communicate

Socio-demographic and familial characteristics are mentioned in relation to the communication concerns of physicians, as previously mentioned, for reasons of language differences, background, relationships between the physicians and patients or familial influence, which can put undue pressure on physicians. Dialogue with the family and patient was often mentioned as a means to find a consensus, even where the patient has little autonomy, as in the case of a child. In this situation, the ability to communicate depends on the way the physicians perceive the child’s autonomy (Table 2/6.20).

### Patient characteristics and treatment characteristics

The patient’s medical characteristics, for example, having a poor prognosis, could be mismatched with treatment characteristics, leading to over- or under-interventionism. The difficulty in judging the situation here is reported as being linked to the subjectivity of the diagnosis and the choice of appropriate treatment. Provided care could be too extreme or, on the contrary, too minimalist, depending on the evaluation of the medical situation, which could be subjective and subject to discussion (Table 2/6.3).

### Mismatched patient characteristics and the physician’s professional situation

Patient characteristics, for example, socio-economic status, could be mismatched with the physician’s professional situation, which could be influenced by costs, claims, lack of time, state of mind, etc. The risk here is also under- or overtreatment, as has been seen with very rich or demanding patients or, conversely, with poor, non-French-speaking patients confronted with physicians who lack enough time to communicate effectively despite the barriers.

Certain professional situations, such as being a senior attending physician, can put physicians at risk of over-treating rich, private patients because they feel obliged to offer something proportionate to justify the patient’s payment (Table 2/6.4).

## Discussion

Several patient characteristics associated with vulnerability were identified. Socio-demographic condition, legal status and financial means seem to be the most important determinants. These characteristics were often linked, as if the costs prevent the system from adapting to the patient’s needs. It has been shown that insurance coverage (more often limited than absent) is the most prevalent pressure identified in the USA [12]. Insurance and the educational status of patients are linked with higher rates of surgical operations [23]. It has been demonstrated that these characteristics (mostly non-medical) influence care through the physician’s communication [24] or perceptions [25, 26].

From the physician’s perspective, decisions are mostly influenced by the patient’s autonomy and medical characteristics, but they are also influenced by medical culture, patients’ families, hospital resources and expensive treatments. Physicians’ feelings (emotional states) can also influence a decision, especially when the situation is sensitive, as has been seen with very ill or demanding patients, putting the patients at risk of being over-treated. Despite guidelines, the fear of missing a lesion played a role in arranging unnecessary examinations [27]. Quality of communication and physician self-awareness are determinants that potentially close these gaps, because vulnerability could appear as a result of communication problems, as has been shown in the literature [28]. Structures provided by the healthcare system are not as diverse as they need to be to cover all situations, and the compensation system is often mentioned as insufficient, especially for uninsured patients [11].

This concept of a dynamic continuum between a patient’s claims and the means to fulfil them has already been described [29]. The gap between claims and means occurs when the processes intended to fulfil these claims are applied to situations in which they are mismatched or insufficient, a situation similar to that which disabled persons face both within the health system and in everyday life [30]. This mismatch can grow with social inequities, working conditions, physicians’ background and the way in which the health system is structured. This concept of vulnerability as a gap or mismatch between the health system’s response to human needs the specific needs of patients that increases the likelihood that these patients will have their interests unjustly considered has important implications across different health systems. We should expect the details and modalities of this phenomenon to change. However, considering these gaps when identifying vulnerable populations is likely to be useful across different systems.

To conclude, our initial definition of vulnerable patients as *those who are more likely to have their interests unjustly considered* was not contradicted by the interviewed physicians, since the gap situations we identified were among the situations in which patients’ interests were not justly considered. On a practical level, however, our results suggest that a more pragmatic approach could complement this more abstract definition of vulnerability. Having categories of ‘situations of mismatch’ is closer to clinical practice in reality. These results add particular situations of mismatch to our definition, providing a more comprehensive approach in the context of the discussion on the limits of patient rights [11]. Bridging these gaps will require additional studies. For patients, it is important to identify the combination of characteristics and situations that put them at risk of being neglected and the skills needed to improve their understanding of a given medical situation. From the physicians’ perspective, clarification is needed in terms of which gaps could be closed by medical or social measures. Situations where too much is done also exist, such as cases of patients who have a high socio-economic status or the capacity to be demanding, and a clarification on how to limit this risk is also needed.

Our study has several limitations. Our findings are situated within a particular context in a given healthcare system (the Swiss one). Generalizations should thus be made only cautiously. However, the experience of resource scarcity is widespread and persistent and makes it likely that similar results may be found elsewhere.

The data collection took place 13 years ago, which means that some of the findings could be different today. We nevertheless believe that our data remain relevant. The main reason is that our findings identify gaps and discrepancies as constitutive of vulnerability and that this is likely to remain the case across different health systems, countries, and time. Moreover, the Swiss health system has not substantially changed since our data were collected. If anything, the system is becoming more expensive, and this has led to a more widespread debate about resource allocation.

The situations mentioned here are linked with equity problems because this was the initial question in the primary study, but vulnerability is not necessarily linked with distributive issues. The mismatch concept based on the gap model is insufficient to identify all potential vulnerability situations. Physicians’ responses can be biased by the fact that they were primarily asked about equity and, second, by the fact that they tend to only remember the most salient situations. Bias due to progressively emerging themes could exist, despite subsequent keyword searches. This is unlikely to have affected the categories, homogenously concentrated in the early and late codes, but the convergence of codes in the matrix may still have been influenced by the absolute quantity of codes because we looked at a convergence density. Our results suggest new hypotheses: Would physicians tell the same stories if they were asked directly about vulnerability? Would the mismatch concept be applicable?

## Conclusions

Our findings suggest that our initial definition of vulnerability as the likelihood of having one’s interests unjustly considered, even if not contradicted by physicians, is not optimal for use in clinical practice because vulnerability is not only linked with patients’ personal characteristics but also with many environmental and personal factors. Vulnerability is a mismatch between the patient’s claims and the services provided, leading to either under- or overtreatment, mainly depending on the socio-economic status of the patient. Physicians express their frustration in such situations because they feel limited when attempting to bridge these gaps. These results provide a more pragmatic way to identify clinical vulnerability based on concrete situations of mismatch. Further studies are necessary to identify patients at risk and the situations where they become vulnerable to provide useful clinical tools to identify these gaps.

## Data Availability

Transcripts of the interviews are available upon request to the authors.

## Abbreviations

LS: Laura Sossauer
MS: Mélinée Schindler
SH: Samia Hurst
